# *Schisandra chinensis* alleviates Hypertriglyceridemia in nonalcoholic fatty liver disease by modulating the gut microbiota and hepatic lipid metabolism: identification of its active fractions

**DOI:** 10.3389/fphar.2025.1715364

**Published:** 2026-01-05

**Authors:** Qi Li, Mei Feng, Qi luo, Chen Yalin Ye, Yi Ke Luo, Yuling Xu

**Affiliations:** Chengdu University, Chengdu, China

**Keywords:** Schisandra chinensis, non-alcoholic fatty liver disease (NAFLD), triglycerides, gut microbiota, lipid metabolism, mendelian randomization

## Abstract

**Background:**

Nonalcoholic fatty liver disease (NAFLD) is a prevalent metabolic disorder characterized by hepatic lipid accumulation and gut microbiota dysbiosis. *Schisandra chinensis (Turcz.) Baill.* (SCH), a traditional hepatoprotective herb, has shown therapeutic potential; however, its mechanisms in NAFLD remain incompletely understood.

**Objective:**

To investigate the protective effects and underlying mechanisms of SCH against NAFLD through integrated genetic, experimental, and multi-omics approaches.

**Methods:**

Mendelian randomization (MR) analysis based on large-scale genome-wide association study (GWAS) datasets was performed to evaluate the causal effects of lipid traits on NAFLD risk. An HFD-induced NAFLD mouse model was used to assess the therapeutic efficacy of SCH extract, with evaluations of serum lipid profiles, liver function, and histopathology. Multi-omics analyses—including 16S rRNA sequencing, untargeted lipidomics, and hepatic metabolite profiling (LC-MS/MS)—were integrated with network pharmacology to predict active metabolite–target–pathway interactions. *In vitro*, an FFA-induced HepG2 steatosis model was used to screen the bioactive fractions of SCH.

**Results:**

SCH significantly reduced hepatic TG accumulation and improved serum lipid profiles. MR analysis confirmed TG as a causal factor for NAFLD. SCH intervention enriched beneficial taxa (e.g., Turicibacter, Muribaculaceae) while suppressing HFD-induced dysbiosis. Lipidomics revealed modulation of glycerophospholipid and choline metabolism. Key phytometabolites (e.g., schisandrin B, gomisin N) were correlated with microbial composition and lipid remodeling. Network pharmacology identified putative targets involved in lipid metabolism, inflammation, and neuroendocrine signaling (e.g., PTGS2, GABRA1, ESR1). GO and KEGG enrichment supported roles in oxidative stress, steroid hormone signaling, and GABAergic synapse pathways, consistent with experimental multi-omics results. *In vitro* assays demonstrated that the n-butanol (BuOH) fraction was the principal bioactive component, significantly reducing lipid accumulation in HepG2 cells.

**Conclusion:**

This integrative study demonstrates that SCH protects against NAFLD by lowering triglycerides, remodeling the gut–liver axis, and reprogramming hepatic lipid metabolism. The BuOH fraction constitutes the main active component, supporting SCH as a promising multi-target candidate for NAFLD therapy.

## Highlights

- Mendelian randomization confirms triglycerides as a causal driver of NAFLD.
- *Schisandra chinensis* (SCH) alleviates NAFLD without affecting food intake.
- SCH reshapes gut microbiota, enriching beneficial genera and suppressing pathogens.
- Hepatic lipidomics shows SCH reprograms glycerophospholipid and sphingolipid pathways.
- Schisandrin B and Gomisin N correlate with microbial shifts and lipid remodeling.
- BuOH fraction identified as the main bioactive extract against hepatic steatosis.

## 1 Introduction

Nonalcoholic fatty liver disease (NAFLD) is a chronic metabolic disorder characterized by abnormal hepatic lipid deposition, which may progress to nonalcoholic steatohepatitis (NASH), fibrosis, and ultimately hepatocellular carcinoma (HCC) (Younossi et al., 2018; Liu et al., 2021; Mao et al., 2024). Dysregulated lipid metabolism, particularly elevated triglyceride (TG) levels, is a hallmark of NAFLD and is closely linked to disease progression (Ding et al., 2023; Rinella et al., 2023). Both clinical and preclinical studies demonstrate that hepatic TG accumulation exacerbates lipotoxic stress, promotes insulin resistance, and accelerates liver injury (Francino, 2015). Thus, targeting TG metabolism has emerged as a promising therapeutic strategy for NAFLD.

To address the long-standing debate over whether elevated TG levels are causally implicated in NAFLD rather than merely associated, we performed Mendelian randomization (MR) analysis using large-scale GWAS data (Semova and Biddinger, 2021; Yazdani et al., 2022; Kyhl et al., 2025; Vabistsevits et al., 2025). Our findings confirmed TG elevation as a causal risk factor for NAFLD, thereby strengthening the theoretical rationale for TG-targeted interventions. This genetic evidence complements multi-omics findings and supports the mechanistic role of *Schisandra chinensis* (SC) in alleviating hepatic steatosis via TG reduction (Burgess et al., 2015; Yazdani et al., 2022).

Recently, the gut microbiota has gained attention as a key regulator of lipid homeostasis through the gut–liver axis. It influences fatty acid absorption, short-chain fatty acid (SCFA) production, and bile acid metabolism (Le Roy et al., 2013; O'Connor et al., 2014; Samuel and Shulman, 2016). Dysbiosis in NAFLD is characterized by reduced beneficial taxa, expansion of pathobionts, and decreased microbial diversity, which exacerbate disease progression by increasing gut permeability and systemic inflammation (Boursier et al., 2016; Tilg et al., 2016; Lu et al., 2025). Restoring gut microbial structure and metabolic function is therefore emerging as a novel therapeutic avenue.

*Schisandra chinensis (Turcz.) Baill.* [Schisandraceae; Schisandrae chinensis fructus], a traditional medicinal and edible herb, contains lignans, alkaloids, and organic acids, and has been reported to exert hepatoprotective, lipid-lowering, antioxidant, and microbiota-modulating effects, potentially through AMPK and PPAR signaling (Zhang M. et al., 2018; Yan et al., 2022; Ehambarampillai and Wan, 2025). Beyond liver disease, SC has shown therapeutic potential in diabetes (Bao et al., 2022), Alzheimer’s disease (Suman Ghosh et al., 2025), and lung cancer (Kong et al., 2023). However, whether SC alleviates NAFLD through a systemic “gut microbiota–metabolite–lipid” axis remains insufficiently understood.

Here, we employed an integrated multi-omics approach to elucidate the mechanism of SC in NAFLD. A high-fat diet–induced NAFLD mouse model was established and treated with aqueous SC extract. MR analysis first verified TG as a causal driver of NAFLD. Subsequently, 16S rRNA sequencing, untargeted hepatic lipidomics, and metabolomic profiling were performed to dissect microbiota, lipid, and phytometabolite signatures. Network pharmacology was further applied to integrate SC component data with target prediction and functional enrichment. *In vitro* experiments confirmed that the n-butanol fraction constitutes the principal bioactive extract, reducing lipid accumulation in HepG2 cells. Collectively, this multi-layered strategy delineates a “gut microbiota–metabolite–lipid” regulatory axis, providing novel mechanistic insights into the hepatoprotective role of SC within the gut–liver metabolic network.

## 2 Materials and methods

### 2.1 Materials and reagents

A high-fat diet (20.11% protein, 60.19% fat, and 19.7% carbohydrate; Cat.No. D12492,Lot No.202403A) was obtained from XIETONG Pharmaceutical Bio-Engineering Co., Ltd. (Jiangsu, China). Reagent kits for measuring alanine aminotransferase (ALT), aspartate transaminase (AST), total cholesterol (TC), triglycerides (TG), low-density lipoprotein cholesterol (LDL-C), and high-density lipoprotein cholesterol (HDL-C) were purchased from Mindray Bio-Medical Electronics Co., Ltd. (Shenzhen, China; Cat.Nos.ALT-100,AST-100,TC-100, TG-100,LDL-100,HDL-100; Lot Nos. M20231201–M20231209). Oil Red O solution was obtained from Solarbio Life Sciences (Beijing, China; Cat.No.G1262, Lot No. 20240105). Fenofibrate tablets were purchased from Hengshan Pharmaceutical Co., Ltd. (Shanghai, China; Cat. No. FBF610, Lot No. 20231210). Dulbecco’s Modified Eagle Medium (DMEM) was purchased from Gibco Life Technologies Co., Ltd. (Guangzhou, China; Cat. No. 11965092, Lot No. 2465763), and fetal bovine serum (FBS; Cat. No. 04-001-1ACS, Lot No. 2304001) was obtained from Biological Industries (Berlin, Germany). Penicillin/streptomycin (100×; Cat. No. BL505A,Lot No. 20231206) was purchased from Biosharp Life Sciences Co., Ltd. (Beijing, China). The Cell Counting Kit-8 (CCK-8; Cat. No. C0038, Lot No. 20240109) was obtained from Beyotime Biotechnology Co., Ltd. (Shanghai, China). Sodium palmitate (Cat. No. KTC1270, Lot No. 20231125) and sodium oleate (Cat. No. KTC1282, Lot No. 20231130) were purchased from KunChuang Technology Development Co., Ltd. (Xi’an, China). UPLC column (C18; 250 mm*4.6 mm,5um), Chromatographic-grade acetonitrile (Cat. No. 1000292501), methanol (Cat. No. 1060352500), and isopropanol (Cat. No. 1096342500) were purchased from Merck (Darmstadt, Germany), while formic acid (Cat. No. F112063, Lot No. 20231215) was obtained from Aladdin (Beijing, China). Ammonium formate (Cat. No. 70221, Lot No. SLBW2981) and methyl tert-butyl ether (MTBE; Cat. No. 34875, Lot No. MKBS1052) were purchased from Sigma-Aldrich (St. Louis, MO, USA). LC-MS-grade water (Cat. No. LC6001, Lot No. 20231225) was purchased from Wahaha Co., Ltd. (Hangzhou, China). Triglyceride (TG) and total cholesterol (TC) detection kits were obtained from Jiancheng Bioengineering Institute (Nanjing, China; Cat. Nos. A110-one to one, A111-1-1; Lot Nos. 20240101–20240103). Polyvinylidene fluoride (PVDF) membranes (Cat. No. IPVH00010, Lot No. R8ED67251) were obtained from Millipore (Burlington, MA, USA).All other reagents were of analytical or LC–MS grade.

### 2.2 Extraction and fractionation

A total of 2.0 kg of dried *S. chinensis* fruits (The plant material was identified and confirmed by Professor Liu Tao of Chengdu University. The specimen (accession number SC 2023-045) has been deposited in the Herbarium of Chengdu University (Chengdu, China). The fruit was collected from Jian’an County, Jilin Province, in October 2023.) were soaked in 20 L of water (10× volume) for 30 min,decocted for 60 min,and re-extracted with an additional 20 L of water for 40 min. The combined decoctions were filtered, concentrated under reduced pressure, and lyophilized to obtain the aqueous extract (yield: 8.35%, moisture content <8%), which was used for animal studies. For fractionation, the concentrated extract was suspended in water and successively partitioned with equal volumes of water-saturated petroleum ether (PET; Lot No. 20231228), ethyl acetate (ETA; Lot No. 20231229), and n-butanol (BuOH; Lot No. 20231230), leaving the residual aqueous fraction (RE). Each fraction was evaporated under reduced pressure, lyophilized, and stored for subsequent cell-based experiments.Fraction yields were as follows: PET 0.78%, EtOAc 1.15%, BuOH 2.64%, and RE 4.21%.Residual solvent levels were within the acceptable limits specified in the Chinese Pharmacopoeia (2020).

### 2.3 Mendelian randomization analysis

Mendelian randomization (MR) was conducted to evaluate the causal effects of lipid traits on NAFLD. GWAS summary statistics for low-density lipoprotein cholesterol (LDL-C), high-density lipoprotein cholesterol (HDL-C), and triglycerides (TG) were retrieved from large-scale public datasets, while NAFLD data were derived from a meta-analysis of six independent cohorts including 77,018 cases and 843,348 controls. Instrumental variables (IVs) were SNPs strongly associated with lipid traits (P < 5.0 × 10^−8^), pruned for linkage disequilibrium (*r* ^2^ < 0.001, >1000 kb) (Yuan et al., 2021). IV strength was assessed by F-statistics (>10) and explained variance (R^2^). Univariable MR (UVMR) estimated individual causal effects, while multivariable MR (MVMR) adjusted for correlations among lipid traits.The inverse variance–weighted (IVW) method served as the primary estimator, complemented by MR-Egger regression and the weighted median approach for sensitivity analyses (Wu et al., 2024). Horizontal pleiotropy was tested using the MR-Egger intercept, outliers were identified and corrected via MR-PRESSO, heterogeneity was evaluated using Cochran’s Q statistic, and leave-one-out analysis assessed single-SNP influence. Analyses were performed in R (v4.4.1) using the “TwoSampleMR,” “MendelianRandomization,” and “MRPRESSO” packages (Wu et al., 2024).

### 2.4 Animal experiment design

Male SPF-grade C57BL/6 mice (8 weeks old) were housed under pathogen-free conditions (22 °C–25 °C, 12 h light/dark cycle). After 1 week of acclimatization, all groups except the control were fed an HFD (protein 20.11%, fat 60.19%, carbohydrate 19.7%) for 8 weeks to induce NAFLD (Lu et al., 2019; Yang et al., 2024), followed by 4 weeks of gavage intervention. Mice were randomly assigned to five groups (n = 8 per group) as follows:Normal control (NC): standard diet + saline; Model control (MC): HFD + saline; SCH low-dose (DC): standard diet + *S. chinensis* (SCH) aqueous extract (0.41 g/kg/d, preventive model); SCH high-dose (SCH): HFD + SCH aqueous extract (1.23 g/kg/d); Positive control (FBR): HFD + fenofibrate (61.5 mg/kg/d).

The doses of SCH (0.41 and 1.23 g/kg/d) were derived from clinical equivalent doses using body surface area conversion and further validated by previous pharmacokinetic and safety studies of Schisandra extracts (Liu et al., 2015). The fenofibrate dose (61.5 mg/kg/d) was selected based on dose conversion from human clinical regimens and prior preclinical studies demonstrating lipid-lowering efficacy in HFD-fed mice (Wang et al., 2016; Lu et al., 2019).

Randomization was performed using a computer-generated random number sequence, and investigators responsible for histological and biochemical assessments were blinded to group allocation. Body weight and food intake were recorded weekly throughout the experiment.

Hypertriglyceridemia was defined as a fasting serum triglyceride (TG) concentration exceeding 1.5 mmol/L in HFD-fed mice, as described in previous studies (Ding et al., 2023; Lu et al., 2019). This criterion was used to confirm the successful establishment of the metabolic disorder model.

At the end of the 12-week study, mice were euthanized, and liver, serum, and fecal samples were collected for subsequent analyses. Fresh fecal samples were obtained at week 8 (prior to intervention) and week 12 (end of treatment) for 16S rRNA sequencing.

The dose levels and group sizes were determined based on pharmacodynamic equivalence and toxicological safety data from previous studies. The sample size (n = 8) provided ≥80% statistical power to detect ≥25% differences in triglyceride levels between groups (α = 0.05).

All animal procedures were reviewed and approved by the Institutional Animal Care and Use Committee (IACUC) of Chengdu University (Approval No. CDU2024-ACU-015) and were conducted in accordance with the Guide for the Care and Use of Laboratory Animals (NIH Publication No. 85-23, revised 2011) (Jones-Bolin, 2012).

### 2.5 Histological and biochemical analyses

Serum biochemical parameters, including alanine aminotransferase (ALT), aspartate aminotransferase (AST), triglycerides (TG), total cholesterol (TC), low-density lipoprotein cholesterol (LDL-C), and high-density lipoprotein cholesterol (HDL-C), were analyzed using an automated biochemical analyzer (Mindray BS-240, Shenzhen, China) with manufacturer’s reagents, following the instrument’s standard operating procedures (Xu et al., 2017).

Liver tissues were fixed in 10% neutral-buffered formalin, embedded in paraffin, sectioned (4 μm), and stained with hematoxylin–eosin (H&E) for histopathological evaluation (Ha et al., 2025). Hepatic lipid accumulation was assessed using Oil Red O staining of frozen liver sections, counterstained with hematoxylin, as previously described (Zhang Y. et al., 2018).

Images were captured under a digital light microscope (Nikon Eclipse Ci-L, Japan), and the hepatic lipid droplet area (%) was quantified using Image-Pro Plus 6.0 software.

All quantitative data are presented as mean ± SD. Statistical analysis was performed using one-way ANOVA followed by Tukey’s *post hoc* test, and P < 0.05 was considered statistically significant (Liu et al., 2018).

### 2.6 16S rRNA sequencing and microbiota analysis

Sequencing data were processed with QIIME2. Raw reads were denoised with DADA2 to remove low-quality sequences, correct errors, and eliminate chimeras, generating amplicon sequence variants (ASVs). Taxonomic classification was assigned to ASVs, and relative abundance, α-diversity, β-diversity, and intergroup differences were analyzed. Principal coordinate analysis (PCoA) and hierarchical clustering were applied for dimensionality reduction and visualization. Statistical differences in microbial composition were tested with Adonis and ANOSIM, while differential taxa were identified using LEfSe.

### 2.7 Lipidomics analysis

Lipidomic profiling was performed using a methanol/MTBE extraction method. Plasma samples were mixed with methanol, followed by MTBE and water, vortexing, sonication, and centrifugation (12,000 × g, 4 °C). The upper phase was collected, vacuum-dried, and reconstituted in isopropanol/acetonitrile (9:1). Quality control (QC) samples were prepared by pooling equal aliquots of each sample. Chromatographic separation was conducted on a UPLC system with an ACQUITY™ Premier CSH C18 column (2.1 × 100 mm, 50 °C, 0.3 mL/min). Mobile phases were ACN/H_2_O (3:2, 10 mM ammonium formate, solvent A) and IPA/ACN (9:1, 10 mM ammonium formate, solvent B). Injection volume was 2 μL. Mass spectrometry was performed on a Thermo Q Exactive HF-X with electrospray ionization in both positive and negative modes (scan range 200–1800 m/z). QC samples were interspersed throughout to monitor reproducibility.

### 2.8 Metabolomics analysis

Frozen samples were thawed on ice, vortexed, and extracted with 20% acetonitrile–methanol containing internal standards. After centrifugation (12,000 × g, 4 °C), supernatants were collected, precipitated at −20 °C, and re-centrifuged. Aliquots were transferred into LC–MS/MS vials for analysis. Data preprocessing included Z-score transformation for 16S profiles, internal standard normalization for lipidomics, and log transformation for metabolomics. Differential taxa were analyzed with the Kruskal–Wallis test; differential lipids and metabolites with Student’s t-test. Correlations between gut microbiota, hepatic metabolites, and lipid species were assessed with Spearman’s rank correlation and visualized using Cytoscape 3.9.1. Data were expressed as mean ± SD, and significance was determined by one-way ANOVA with Tukey’s *post hoc* test (P < 0.05). Integration of multi-omics datasets was conducted using DIABLO for biomarker discovery and MMVEC to infer microbe–metabolite associations.

### 2.9 Network pharmacology analysis

Active components of SC were collected from the Traditional Chinese Medicine Systems Pharmacology Database (TCMSP, https://tcmsp-e.com) (Ru et al., 2014) and literature reports, applying oral bioavailability (OB ≥ 30%) and drug-likeness (DL ≥ 0.18) as filters. Additional known bioactives (e.g., schisandrin B, gomisin N) were manually curated. Target prediction was conducted with SwissTargetPrediction (Daina et al., 2019) and BATMAN-TCM (Liu et al., 2016), and standardized using UniProt. NAFLD-related targets were retrieved from GeneCards (Stelzer et al., 2016) and DisGeNET (Pinero et al., 2020). Overlapping targets between SC components and NAFLD were identified as candidate therapeutic targets. Functional enrichment was performed with DAVID (Huang da et al., 2009) for GO and KEGG pathways, visualized in R (clusterProfiler package) (Yu et al., 2012). Component–target–pathway networks were constructed in Cytoscape 3.9.1 (Shannon et al., 2003).

### 2.10 Cell culture and treatments

HepG2 cells were cultured in DMEM supplemented with 10% fetal bovine serum at 37 °C in 5% CO_2_. Cells were thawed, passaged, and cryopreserved following standard procedures. For cytotoxicity and activity screening, 4,000 cells/well were seeded into 96-well plates and treated with varying concentrations of SC fractions (PET, ETA, BuOH, RE). After 24 h, cell viability was assessed with CCK-8 at 450 nm. For the free fatty acid (FFA)-induced steatosis model, HepG2 cells (1.2 × 10^5^/mL) were seeded into 6-well plates and exposed to 0.5 mM FFA for 24 h. Cells were divided into control, model, and treatment groups; treatment groups received SC fractions diluted in FFA-containing medium. Lipid accumulation was visualized by Oil Red O staining, while TG and TC levels were quantified using commercial kits.

### 2.11 Statistical design and data reporting

All statistical analyses were performed using GraphPad Prism 8.0 (GraphPad Software, San Diego, CA, USA) and R software (version 4.4.1).

For *in vivo* experiments, data were expressed as mean ± standard deviation (SD) with a sample size of n = 8 mice per group. For *in vitro* assays, experiments were performed in triplicate (n = 3 biological replicates). The definitions of sample size and error bars have been indicated in all figure legends (Figures 2–8).

Comparisons among multiple groups were conducted using one-way analysis of variance (ANOVA) followed by Tukey’s *post hoc* test to assess pairwise differences. Statistical significance was set as P < 0.05, P < 0.01, and *P < 0.001, respectively.

For multi-omics analyses, including DIABLO (Data Integration Analysis for Biomarker discovery using Latent variable approaches), MMVEC (Microbe–Metabolite Vectors), and Cytoscape-based network construction, the following statistical criteria were applied: false discovery rate (FDR) < 0.05 and absolute correlation coefficient |ρ| > 0.4. Cytoscape networks were generated using n = 30 representative taxa–metabolite pairs, selected from the top 50 features identified by DIABLO.

All analyses were conducted under two-tailed assumptions. No data were excluded unless technical failure was confirmed.

## 3 Results

### 3.1 Triglycerides are a key causal risk factor for NAFLD

**FIGURE 1:**
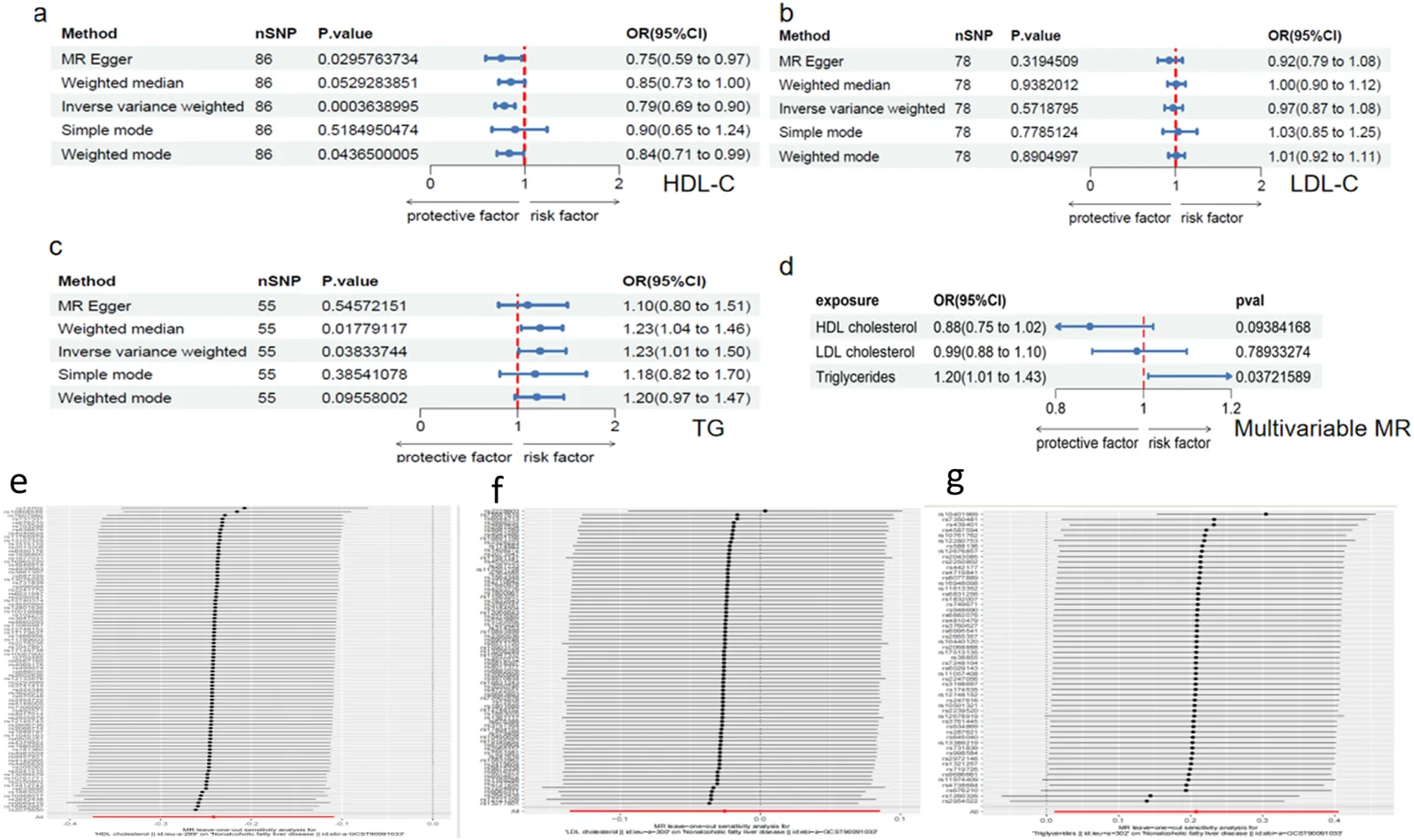
Triglycerides as a causal factor for NAFLD validated by Mendelian randomization. **(a-c)** Forest plots illustrating the causal effects of TG, HDL-C, and LDL-C on NAFLD. **(d)** Multivariable Mendelian randomization (MVMR) analysis. **(e-g)** Leave-one-out sensitivity analysis for Mendelian randomization estimates.

Using genome-wide significance thresholds, we identified 78, 86, and 55 independent SNPs associated with LDL-C, HDL-C, and triglycerides (TG), which together explained 9.53%, 6.41%, and 5.54% of trait variance, respectively. All selected instruments demonstrated adequate strength (F > 10). Univariable MR using the inverse-variance weighted (IVW) estimator revealed a significant positive causal effect of TG on NAFLD risk, a protective effect for HDL-C, and no significant association for LDL-C (MR-Egger: OR = 0.923, P = 0.319) (Figures 1a–c). Multivariable MR (MVMR), adjusting for correlations among lipid traits, confirmed TG as an independent risk factor for NAFLD (OR = 1.189; 95% CI, 1.068–1.324; P < 0.001) and retained a protective effect of HDL-C (OR = 0.792; 95% CI, 0.709–0.883; P < 0.001), whereas LDL-C remained non-significant (Figure 1d). Sensitivity analyses (MR-Egger regression, weighted median, MR-PRESSO, and leave-one-out) produced consistent results and showed no evidence of directional pleiotropy (all pleiotropy tests P > 0.05) (Figures 1e–g). Collectively, these analyses indicate that circulating TG are a critical metabolic determinant and causal driver of NAFLD.

### 3.2 *Schisandra chinensis* ameliorates NAFLD phenotype and lowers circulating TG

**FIGURE 2:**
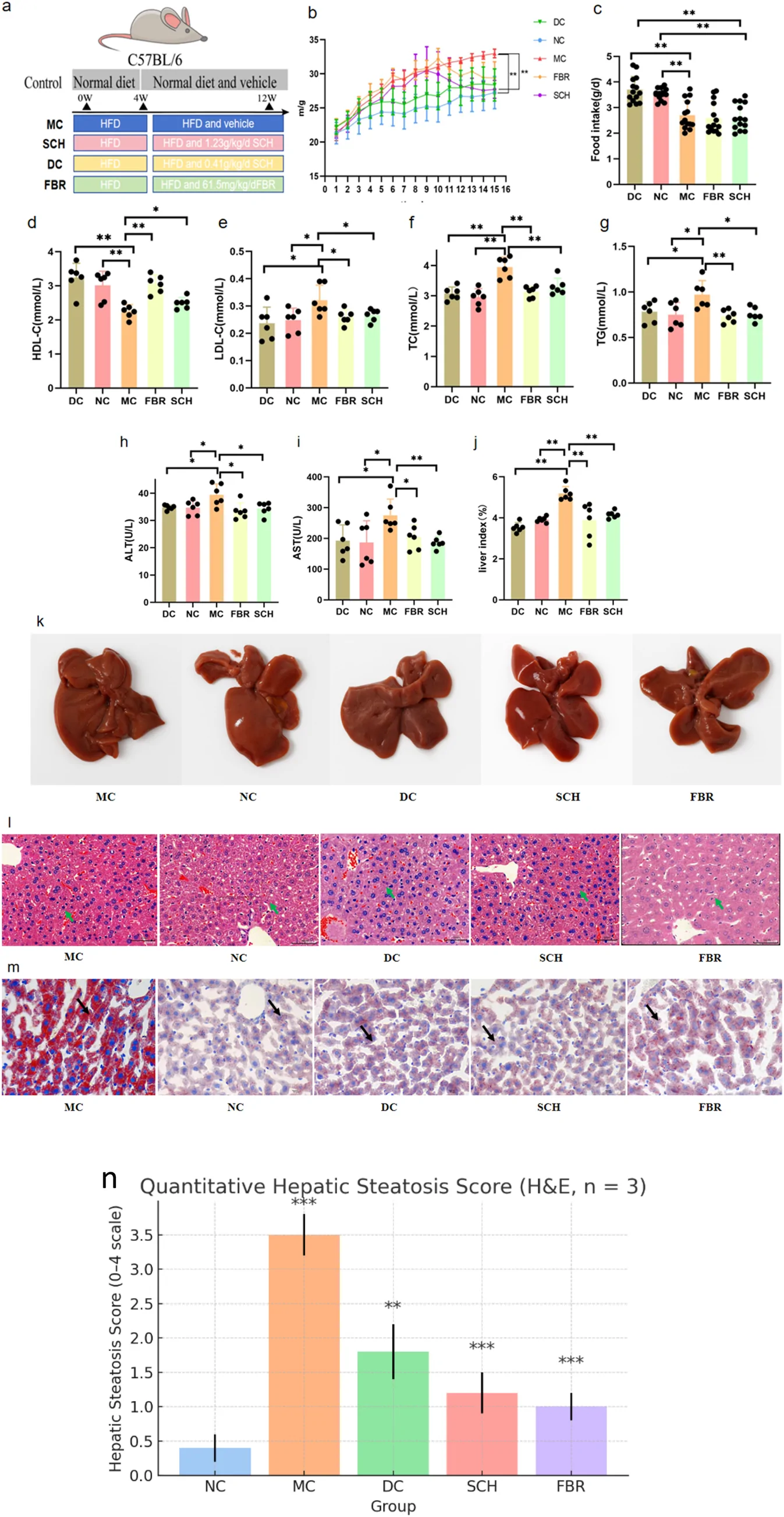
Physiological and histological improvements after *Schisandra chinensis* treatment.Data are expressed as the mean ± SD (n = 8) **(a)** In the animal experiment process, the Control group is represented in gray, the Model group in blue, the SCH group in pink, the DC group in yellow, and the FBR group in green. **(b)** Effect of *Schisandra chinensis* on body weight in NAFLD mice. **(c)** Food intake of each group during intervention. **(d–g)** Changes in serum lipid profiles after intervention. **(h–j)** Improvements in liver function biomarkers. **(k)** Pathological changes in mouse liver tissue. **(l)** H&E staining of liver tissues. **(m)** Oil Red O staining of hepatic lipid droplets. **(n)** quantitative steatosis scores. (0–4 scale; n = 3 per group, mean ± SD). Significance labels in the figure: P < 0.001: MC group vs. NC group, SCH group vs. MC group, FBR group vs. MC group P < 0.01: DC group vs. MC group.

The experimental timeline is shown in Figure 2a. HFD feeding induced significant weight gain versus controls (Figure 2b). Treatment with Schisandra aqueous extract markedly reduced body weight to levels comparable with fenofibrate (FBR) treatment, without altering food intake (Figure 2c), suggesting a metabolic rather than anorectic mechanism. Schisandra substantially improved the serum lipid profile (Figures 2d–g): TG decreased by 44%, while total cholesterol (TC) and LDL-C were significantly reduced and HDL-C increased. Liver function indices also improved (Figures 2h–j), with ALT and AST reduced by 42% and 37%, respectively, and a lower liver index. Histopathology revealed widespread macrovesicular steatosis in model mice (P < 0.01), which was significantly attenuated by Schisandra treatment (P < 0.001), together with preservation of hepatic architecture and a reduction in lipid droplet aggregation (Figures 2k–n; Table 1). These results demonstrate that Schisandra extract effectively mitigates HFD-induced hepatic steatosis, dyslipidemia, and liver injury.

**TABLE 1:** Statistical results of the percentage (%) of area of lipid droplet expression in liver tissues (±SD).

Groups	N	$\bar{x}$ ±SD
NC	3	0.67 ± 0.31
DC	3	1.39 ± 0.99
MC	3	3.21 ± 0.82^**△△△^
SCH	3	1.19 ± 0.34^●●●^
FBR	3	1.06 ± 0.31^●●●^

*P < 0.05 **P < 0.01 ***P < 0.001 for the model alone group compared with the blank control group; ^△^P < 0.05. ^△△^P < 0.01. ^△△△^P < 0.001 for the simple model group compared with the blank administration group; ^●^P < 0.05. ^●●^P < 0.01. ^●●●^P < 0.001 the remaining groups compared with the model alone group.

### 3.3 Schisandra remodels gut microbiota composition

**FIGURE 3:**
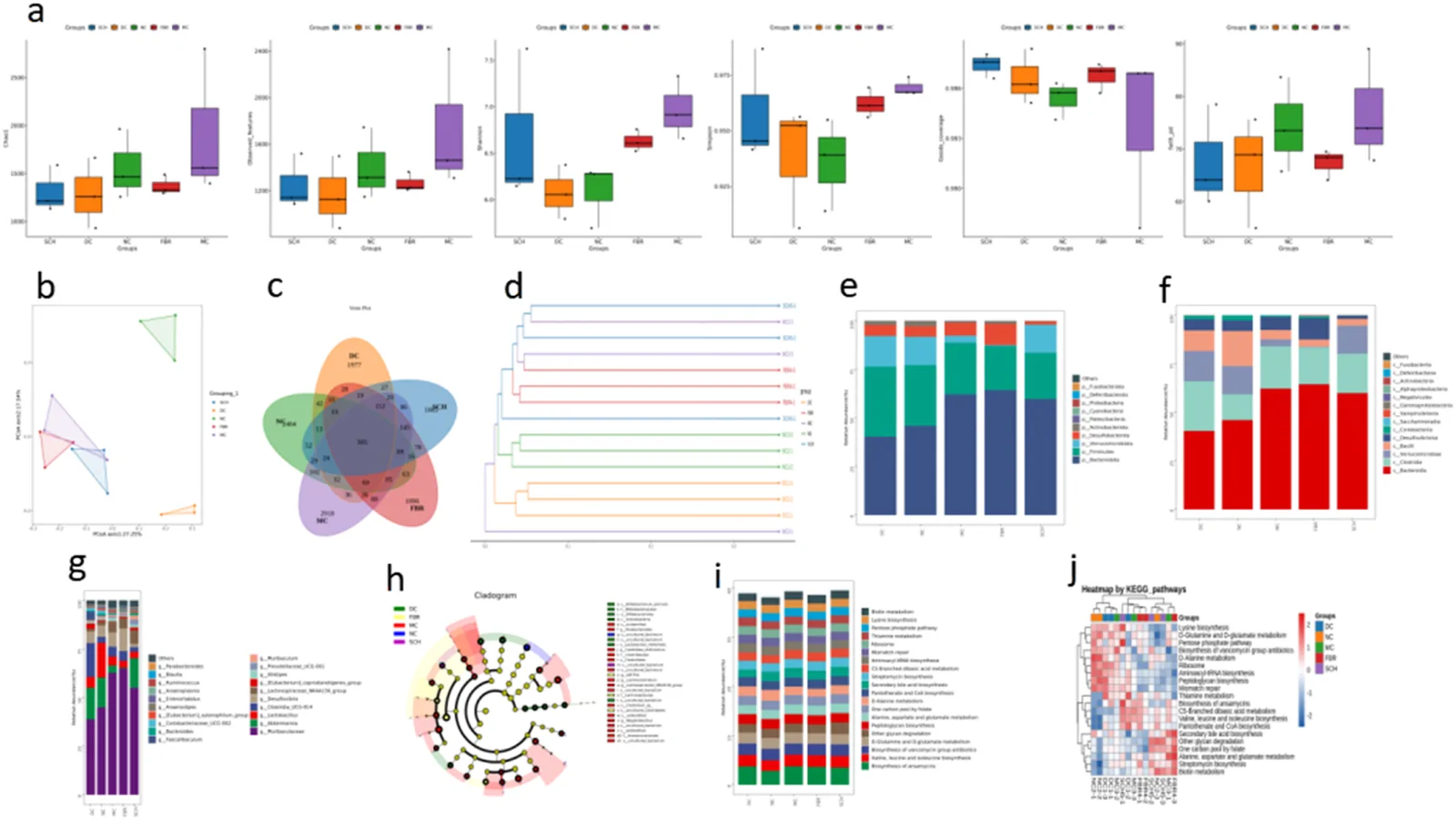
Gut microbiota modulation by *Schisandra chinensis*. **(a)** Alpha diversity of gut microbiota. **(b)** PCoA plot shows clustering differences between groups. **(c)** Venn diagram of ASV distribution. **(d)** Phylogenetic tree indicates microbiota convergence in SCH group toward normal controls. **(e–g)** Taxonomic composition at phylum, class, and genus levels. **(h)** LEfSe analysis identifying key microbial taxa across groups. **(i,j)** Functional predictions of gut microbiota using PICRUSt analysis. Data are expressed as the mean ± SD (n = 8).

High-throughput 16S rRNA sequencing showed pronounced gut microbial alterations in NAFLD mice that were partially restored by Schisandra treatment. NAFLD increased species richness (Chao1, observed features, Faith’s PD; P < 0.05) but reduced evenness (Shannon and Simpson indices), consistent with dysbiosis (Figure 3a). Schisandra reduced excessive richness and improved community evenness toward control levels. Principal coordinate analysis (PCoA) demonstrated clear separation between control and model groups (Figure 3b), while Schisandra-treated samples clustered closer to controls. Taxonomic profiling revealed NAFLD-associated depletion of Firmicutes and enrichment of Bacteroidetes, resulting in a decreased F/B ratio that was reversed by Schisandra (Figures 3c,d). At phylum and genus levels (Figures 3e–g), beneficial taxa (e.g., members of Clostridia, *Enterococcus*, *Lactobacillus*, and rumen-associated genera) were enriched after treatment, whereas pro-inflammatory taxa (e.g., Proteobacteria, Desulfovibrio, Akkermansia in this dataset) were suppressed. LEfSe analysis corroborated enrichment of inflammatory taxa in NAFLD and their attenuation by Schisandra (Figure 3h). Functional prediction (PICRUSt/KEGG) indicated NAFLD-associated enrichment in lipid-related pathways, including bile acid and SCFA metabolism, which were significantly downregulated following Schisandra treatment (Figures 3i,j). Together, these data indicate that Schisandra alleviates NAFLD in part by reshaping gut microbial ecology and restoring gut–liver metabolic functions.

### 3.4 Hepatic lipidome is substantially reprogrammed by schisandra

**FIGURE 4:**
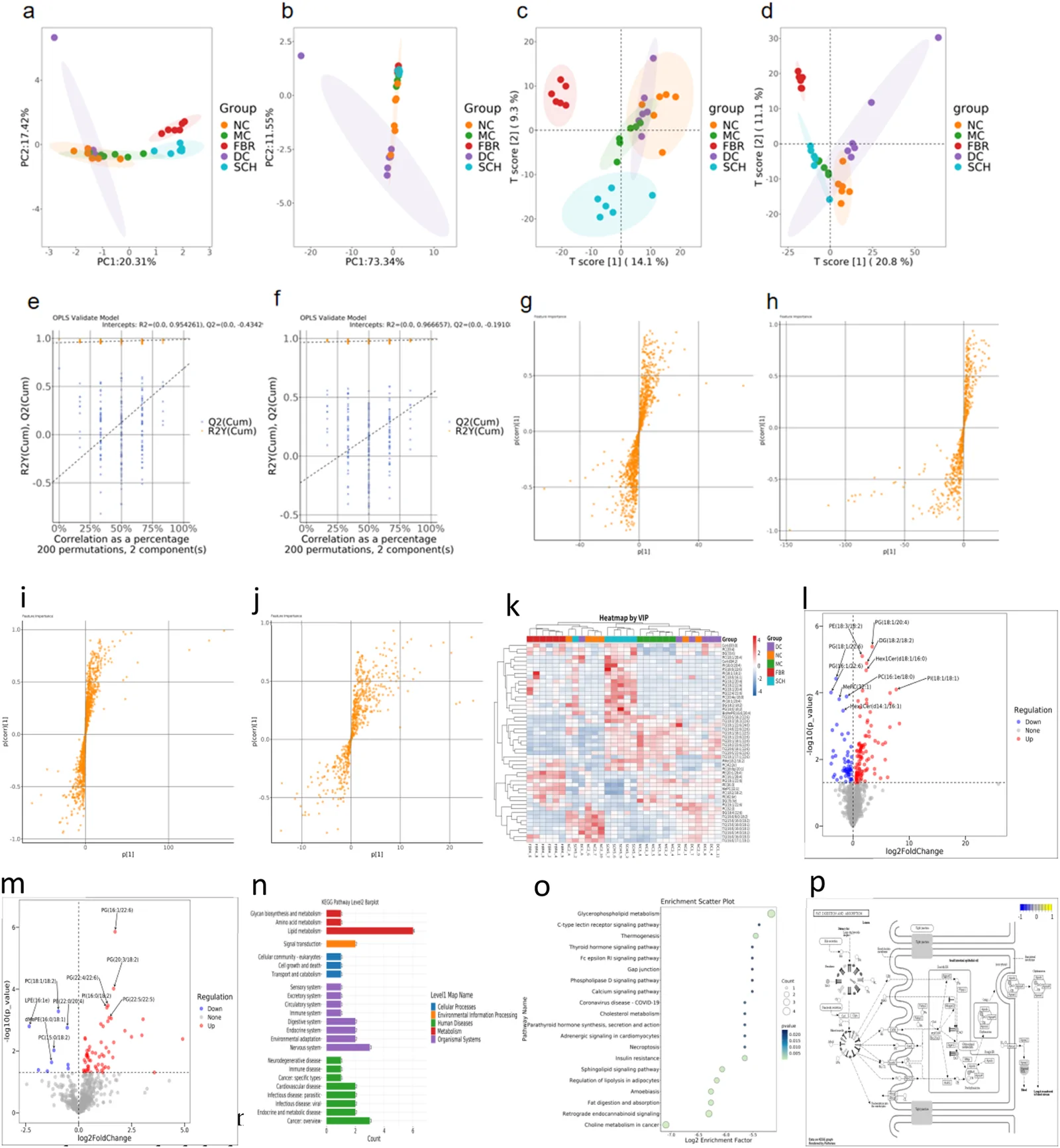
**(a,b)** Principal component analysis (PCA) plots under positive and negative ion modes reveal global metabolic shifts among the groups. **(c,d)** Partial least squares discriminant analysis (PLS-DA) shows clear separation between *Schisandra chinensis* (SCH)-treated mice and the model group (MC), indicating treatment-induced lipidomic alterations. **(e,f)** OPLS-DA permutation tests (200 iterations) validate the robustness and predictive power of the models, as evidenced by negative Q^2^ intercepts and non-overfitted R^2^ values. **(g-j)** S-plot analysis identifies key metabolites contributing to group separation in positive and negative ion modes. **(k)** Heatmap illustrating the relative abundance of key lipid metabolites identified by variable importance in projection (VIP) scores from PLS-DA analysis. **(l,m)** Volcano plots illustrate the distribution of significantly altered hepatic lipid species between the *Schisandra chinensis* (SCH) group and the model control (MC) group under positive (B) and negative (C) ion modes. Each dot represents a lipid metabolite.** (n-p)** Lipid pathway enrichment and metabolic network. Data are expressed as the mean ± SD (n = 8).

Untargeted LC–MS lipidomics of liver tissue in both ion modes revealed pronounced intergroup differences. PCA and PLS-DA demonstrated that the SCH group separated distinctly from the MC group and shifted closer to NC and FBR groups (Figures 4a–d), indicating partial restoration of the lipidomic profile. Model validity was confirmed by 200-permutation tests (Q^2^ intercept <0), excluding overfitting (Figures 4e,f). S-plot and VIP analysis identified 50 representative differential lipids (Figures 4g–k), including phosphatidylcholines (PC), phosphatidylethanolamines (PE), and multiple TG species that were significantly downregulated in SCH mice. Volcano plots highlighted numerous up- and downregulated features (Figures 4l,m); notably, PG (18:2/18:2), PC(16:1e/18:0), and PE (18:3/18:2) were markedly decreased, while certain phosphatidylglycerols and monoacylglycerols were restored. KEGG enrichment implicated glycerophospholipid, glycerolipid, sphingolipid, choline, and arachidonic acid metabolism (Figures 4n,o), pathways relevant to lipid droplet formation, membrane integrity, and inflammation. Network analyses identified TG (18:1/18:2/23:1), LPC(18:0), and Cer(d18:1/16:0) as central nodes. Gene expression/pathway visualization showed upregulation of fatty acid uptake and re-esterification genes (Cd36, Slc27a1, Mogat2, Fabp1/2, Got2) and downregulation of cholesterol transporters (Scarb1, Npc1l1) (Figure 4p), implying Schisandra reprograms hepatic and intestinal lipid handling to ameliorate NAFLD.

### 3.5 Secondary metabolomics identifies schisandra-derived bioactive metabolites linked to lipid regulation

**FIGURE 5:**
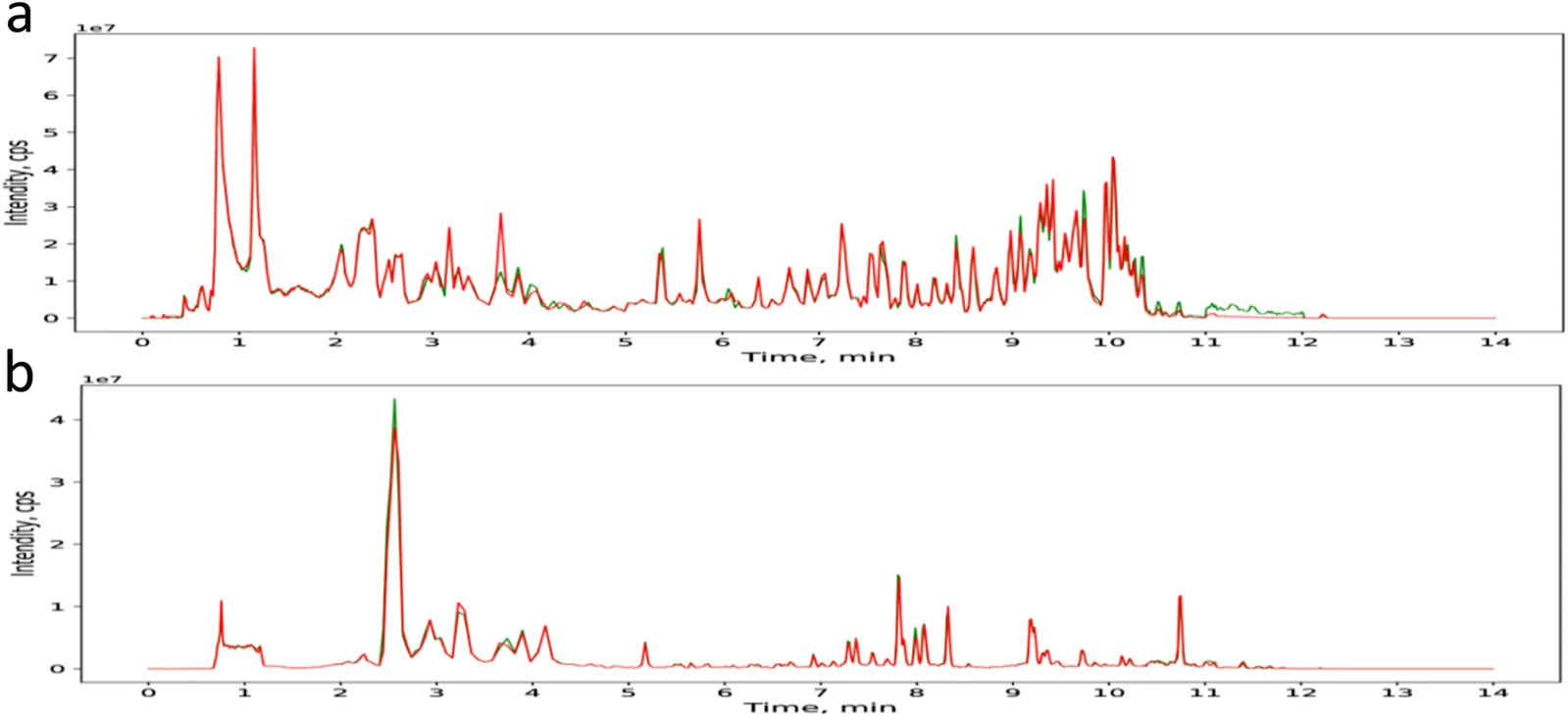
TIC of the components of SCH in positive and negative ion modes. **(a)** TIC of the components of SCH in positive ion mode. **(b)** TIC of the components of SCH in negative ion mode.

**FIGURE 6:**
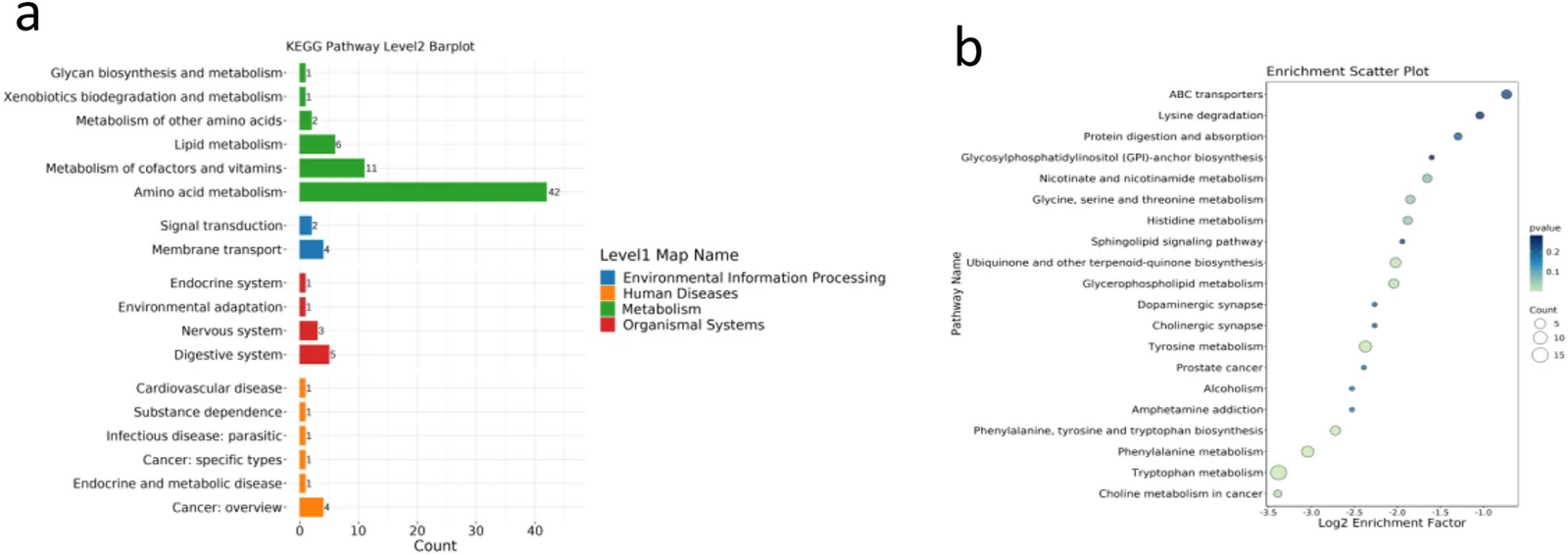
**(a,b)** KEGG enrichment of S. chinensis-derived metabolites.Metabolites were enriched in lipid-related pathways such as choline metabolism and fatty acid degradation. Data are expressed as the mean ± SD (n = 8).

Non-targeted secondary metabolomics (LC–MS) of SCH group livers identified 46 differential secondary metabolites, 17 of which were confirmed as Schisandra-derived (Figure 5; Table 2), including lignans (schisandrin A/B/C, gomisin D/G/N), triterpenoids (schisphenol, schisantherin methyl), and flavonoid/organic acids (quercetin, gallic acid). These metabolites were significantly enriched in SCH livers, indicating substantial absorption and hepatic bioavailability; schisandrin B and gomisin N were the most abundant. KEGG enrichment (Figures 6a,b) mapped these phytometabolites to glycerophospholipid metabolism, choline metabolism in cancer, fatty acid degradation, and bile secretion—pathways directly involved in TG synthesis, transport, and catabolism. Correlative analyses suggested negative associations between schisandrin C and intermediates such as glycerol-3-phosphate and CDP-choline, implicating potential modulation of key enzymes (e.g., GPAT, CPT) that could suppress TG synthesis or enhance degradation. Likewise, gomisin G and schisphenol were enriched in choline and pantothenic acid metabolism, suggesting roles in phospholipid biosynthesis and acetyl-CoA generation to maintain lipid homeostasis.

**TABLE 2:** 17 metabolites identified and characterized in *Schisandra chinensis* fruits by UPLC-Q-TOF/MS.

NO.	Metabolites	Class II	Molecular formula	Adduct	Molecular weight (Da)
1	Schizandrin A	Lignans	C_24_H_32_O_7_	[M + H]^+^	432.2148
2	Schizandrin B	Lignans	C_23_H_28_O_7_	[M + Na]^+^	439.1715
3	Schizandrin C	Lignans	C_22_H_24_O_6_	[M + H]^+^	384.1573
4	Gomisin A	Lignans	C_23_H_28_O_7_	[M + H]^+^	416.1835
5	Gomisin D	Lignans	C_28_H_34_O_10_	[M + Na]^+^	553.2042
6	Gomisin G	Lignans	C_30_H_32_O_9_	[M + Na]^+^	559.1933
7	Gomisin N	Lignans	C_23_H_28_O_6_	[M + H]^+^	401.1959
8	Gomisin B	Lignans	C_28_H_34_O_9_	[M + H]^+^	514.2203
10	Schisandrin A*	Triterpenoid saponins	C_24_H_32_O_6_	[M + H]^+^	416.2199
11	Schisandrone	Lignans	C_21_H_24_O_5_	[M-H]^-^	356.1624
12	Quercetin	Flavonols	C_15_H_10_O_7_	[M + H]^+^	302.0427
13	Gallate	Phenolic acids	C_7_H_6_O_5_	[M-H]^-^	170.0215
14	Deoxygomisin A	Lignans	C_23_H_28_O_6_	[M + H]^+^	400.1886
15	Schisantherin D	Lignans	C_29_H_28_O_9_	[M + Na]^+^	520.1733
16	Schisantherin P	Lignans	C_22_H_24_O_8_	[M + H]^+^	416.1471
17	Schisantherin L	Lignans	C_27_H_30_O_9_	[M + H]^+^	498.189

### 3.6 Multi-omics integration reveals a “microbiota–metabolite–TG” regulatory axis for schisandra action

**FIGURE 7:**
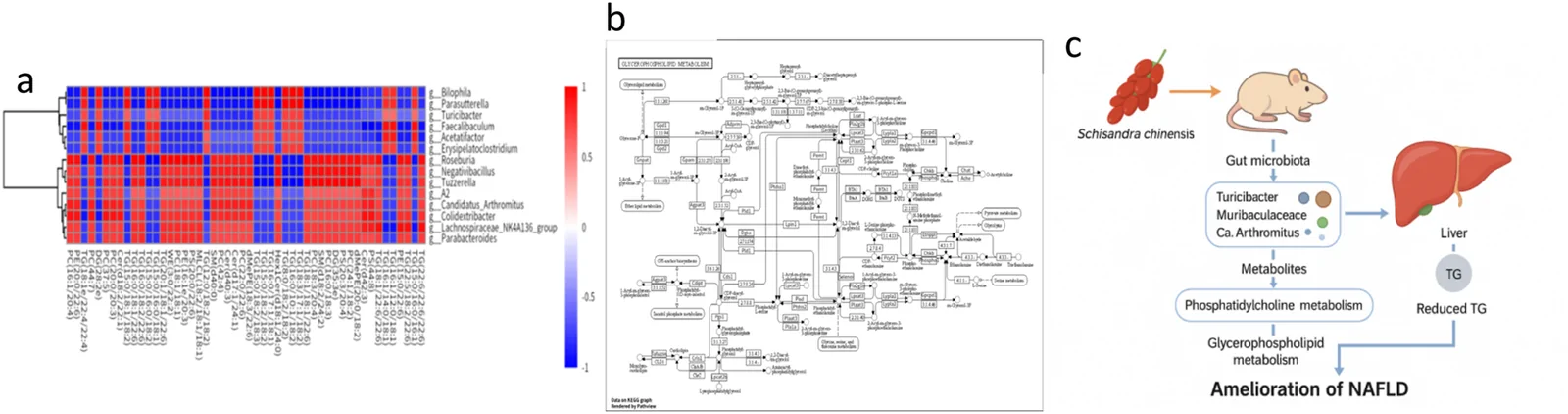
**(a)** Spearman correlation heatmap between hepatic triglyceride species and gut microbial genera. **(b)** Multi-omics interaction network. **(c)** Proposed mechanistic model of *Schisandra chinensis* in NAFLD improvement. Data are expressed as the mean ± SD (n = 8).

To elucidate multilayered mechanisms, we integrated microbiome, lipidome, and secondary metabolome datasets and constructed a comprehensive interaction network. Spearman correlation analysis (Figure 7a) showed that hepatic TG levels were significantly negatively correlated with several beneficial genera (e.g., Muribaculaceae, Candidatus Arthromitus, Turicibacter) (ρ = −0.42 to −0.68, P < 0.05), and positively correlated with inflammatory taxa (e.g., Desulfovibrio, Lachnospiraceae_NK4A136). Multiple TG species (e.g., TG (16:0/18:1/22:6)) and ceramides (Cer(d18:1/24:1)) were negatively correlated with Schisandra signature metabolites (schisandrin B, gomisin N). Cross-omics network analysis (Figure 7b) demonstrated coordinated links whereby beneficial taxa associated positively with Schisandra lignans and inversely with hepatotoxic TG species, while pathobionts correlated positively with sphingolipids and lipid droplet–associated TGs. Shared pathway enrichment highlighted glycerophospholipid, sphingolipid, choline metabolism, and secondary bile acid synthesis as convergent hubs across PICRUSt predictions, lipidomics, and phytometabolite mapping. Enzyme-centric inference implicated hepatic GPAT and LPCAT as plausible targets of Schisandra lignans. Based on these data, we propose a dual mechanism in which Schisandra modulates the gut microbiota (indirect route) and delivers bioactive lignans to the liver (direct route) to suppress choline oxidation and lipid droplet biogenesis, thereby reducing TG accumulation and ameliorating NAFLD. The integrated “microbiota–metabolite–lipid” network model is summarized in Figure 7c.

### 3.7 Network pharmacology reveals multi-target actions of schisandra

**FIGURE 8:**
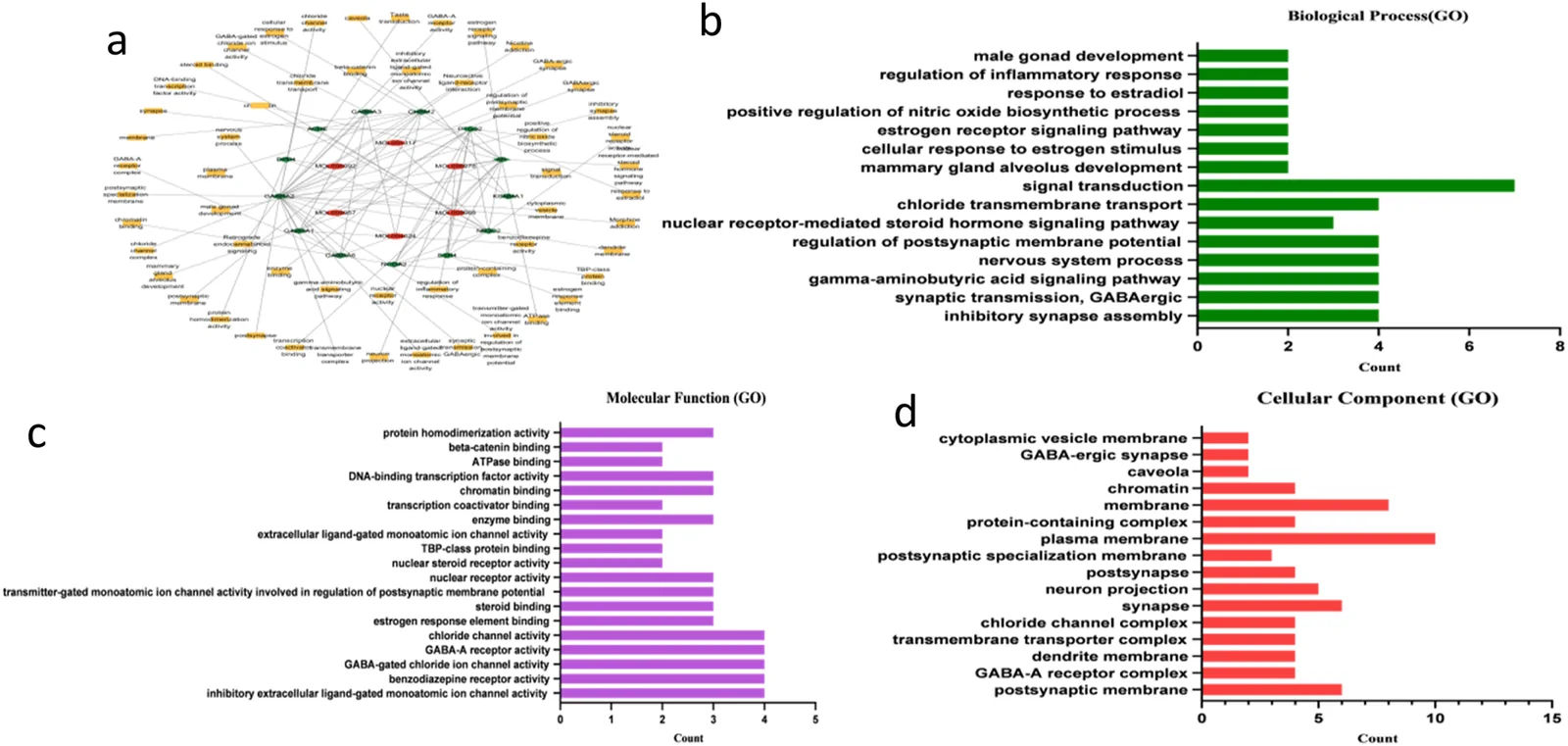
**(a)** Metabolite–target–pathway interaction network constructed using Cytoscape. Red nodes represent active metabolites from *Schisandra chinensis*, green nodes represent predicted protein targets, and yellow nodes represent enriched GO terms or KEGG pathways. **(b–d)** Gene Ontology (GO) enrichment bar plots illustrating the top 10 enriched terms in biological process (BP), cellular component (CC), and molecular function (MF) categories.Bubble plot summarizing GO and KEGG pathway enrichment results for S. chinensis-related targets.

Network pharmacology analysis of 20 representative Schisandra metabolites (including schisandrin B, gomisin N, and schisantherin methyl) predicted 146 putative protein targets via SwissTargetPrediction and BATMAN-TCM. GO and KEGG enrichment (visualized in Cytoscape; Figures 8a–d) indicated that these targets converge on neurotransmitter receptor subunits (e.g., GABRA1/2), nuclear receptors (e.g., ESR1), and inflammatory mediators (e.g., PTGS2). Enriched pathways included neuroactive ligand–receptor interaction, steroid hormone signaling, and GABAergic synapse. These results suggest that Schisandra’s lipid-lowering and hepatoprotective effects may involve not only direct modulation of lipid metabolism but also neuroendocrine and immune regulatory mechanisms, providing a systems-level rationale for its traditional use.

### 3.8 n-Butanol fraction (BuOH) constitutes the principal anti-steatotic bioactivity *in vitro*

**FIGURE 9:**
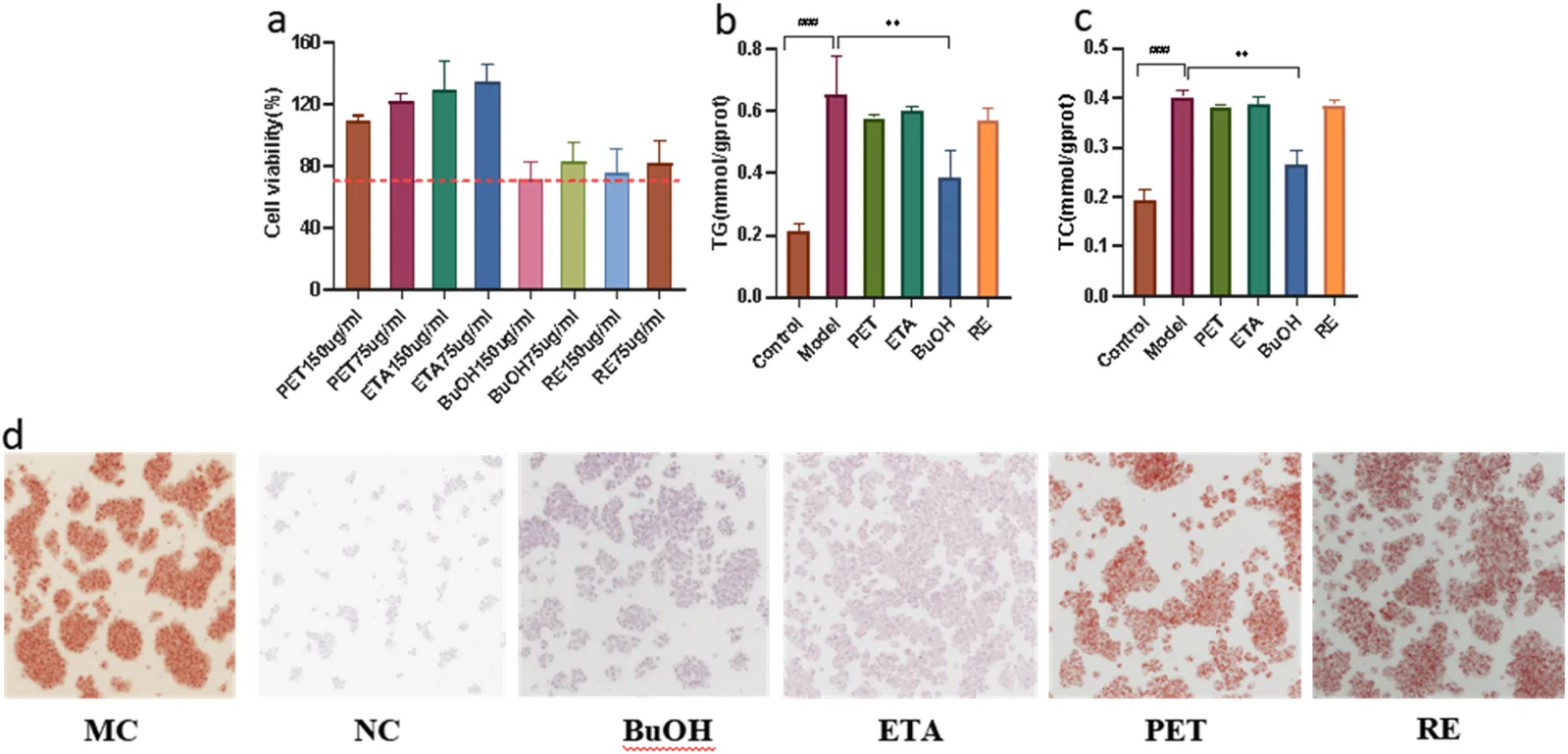
**(a)** Cell viability; **(b)** TG levels; **(c)** TC levels; **(d)** Representative microscopy images.

To identify the most active fraction, HepG2 cells with FFA-induced steatosis were treated with PET, ETA, BuOH, and residual (RE) fractions. Cytotoxicity assays showed no significant viability loss for all fractions at 75 μg/mL (Figure 9a), whereas BuOH at 150 μg/mL reduced viability below 80%. Oil Red O staining confirmed robust lipid accumulation in the model. At 75 μg/mL, BuOH markedly decreased intracellular lipid droplet accumulation relative to PET, ETA, and RE fractions, which were largely inactive (Figure 9d). Biochemical assays corroborated these observations: BuOH treatment significantly reduced cellular TG and TC levels (P < 0.05) while other fractions showed no activity (Figures 9b,c). These data identify the BuOH fraction as the principal *in vitro* bioactive fraction mediating anti-steatotic effects of Schisandra.

## 4 Discussion

This study employed an integrated research strategy combining Mendelian randomization (MR), *in vivo* and *in vitro* experiments, gut microbiota analysis, hepatic lipidomics, secondary metabolomics, and network pharmacology to elucidate the protective mechanisms of *Schisandra chinensis* (SCH) against nonalcoholic fatty liver disease (NAFLD). The multi-layered approach enabled comprehensive insight into both systemic and molecular processes involved in SCH-mediated hepatoprotection.

MR analysis based on large-scale GWAS datasets confirmed that circulating triglycerides (TG) are an independent causal risk factor for NAFLD, while high-density lipoprotein cholesterol (HDL-C) exerts a protective effect and low-density lipoprotein cholesterol (LDL-C) shows no significant association. These findings are consistent with previous reports linking dyslipidemia to NAFLD pathogenesis (Pang et al., 2022; Su et al., 2025), reinforcing the rationale for TG-lowering interventions as a key therapeutic strategy.

In the high-fat diet (HFD)-induced NAFLD mouse model, SCH markedly mitigated weight gain, improved serum lipid profiles, and alleviated hepatic injury without altering food intake, indicating a metabolic regulatory effect rather than appetite suppression. Histological and biochemical analyses revealed that SCH significantly reduced hepatic lipid deposition and steatosis severity, achieving improvements comparable to those of fenofibrate. These results align with previous evidence of SCH’s lipid-lowering and hepatoprotective properties (Liu et al., 2015; Zheng et al., 2024).

Microbiome profiling revealed that NAFLD was associated with increased microbial richness, decreased evenness, and enrichment of pro-inflammatory taxa such as Desulfovibrio and Akkermansia. SCH treatment partially reversed these dysbiotic changes by enhancing beneficial taxa (e.g., *Lactobacillus*, Ruminococcus) and restoring microbial community balance. Functional prediction further indicated that SCH modulated bile acid and short-chain fatty acid (SCFA) pathways—both central to gut–liver metabolic communication. These findings support the concept that SCH alleviates NAFLD partly through remodeling the gut–liver axis, consistent with prior studies on microbiota-mediated liver injury (Lopez et al., 2012; Wang et al., 2016).

Comprehensive hepatic lipidomic analysis demonstrated that SCH reprogrammed lipid metabolism by downregulating phosphatidylcholines (PC), phosphatidylethanolamines (PE), and multiple TG species, while restoring specific phosphatidylglycerols (PG) and monoacylglycerols toward normal levels. KEGG enrichment implicated glycerophospholipid, glycerolipid, and sphingolipid metabolism—key pathways regulating lipid droplet formation, membrane integrity, and inflammation. The identification of hub lipids such as TG (18:1/18:2/23:1), LPC(18:0), and Cer(d18:1/16:0) underscores a coordinated lipid network modulation, providing mechanistic insight into how SCH mitigates hepatic lipid overload.

Secondary metabolomic profiling revealed significant hepatic accumulation of SCH-derived lignans (e.g., schisandrin B, gomisin N), which correlated strongly with specific microbial taxa and lipid species, forming a “microbiota–metabolite–lipid” interaction network. These bioactive phytometabolites are known to activate AMPK and PPARα pathways, promoting fatty acid oxidation and suppressing *de novo* lipogenesis (Yan et al., 2022). Network pharmacology further identified potential molecular targets—such as PTGS2, ESR1, and GABA receptor subunits—connecting lipid metabolism, inflammation, and neuroendocrine signaling. This systems-level coherence among lipidomics, metabolomics, and computational pharmacology provides robust mechanistic evidence that SCH exerts multi-target, cross-organ regulation.

*In vitro* studies using FFA-induced HepG2 steatosis models confirmed that the n-butanol (BuOH) fraction exhibits the strongest anti-steatotic activity, significantly reducing intracellular lipid droplets and decreasing cellular TG and TC levels, while PET, ETA, and RE fractions showed limited effects. This aligns with prior chemoprofiling reports indicating that BuOH fractions are enriched in lignans such as schisandrins and gomisins, which are key hepatoprotective and lipid-lowering metabolites (Liu et al., 2015).

Collectively, these multi-omics and experimental findings support a coherent mechanistic model in which SCH ameliorates NAFLD through three complementary routes: 1. Systemic metabolic modulation–validated by MR and *in vivo* results confirming TG lowering as a causal therapeutic axis; 2. Gut–liver axis regulation–evidenced by microbiota restoration and metabolite–lipid interactions; 3. Direct hepatic reprogramming–driven by absorbable phytometabolites that regulate lipid metabolism and suppress lipid droplet biogenesis.

Together, these synergistic effects position SCH as a promising multi-target botanical candidate for NAFLD prevention and therapy.

Despite the integrative multi-omics and experimental approach, Despite the integrative multi-omics and experimental approach, several limitations should be acknowledged.

- The causal roles of specific microbial taxa and metabolites remain to be validated through fecal microbiota transplantation or monoassociation experiments.
- Direct biochemical confirmation of key hepatic enzyme targets (e.g., GPAT, LPCAT) requires additional genetic or pharmacologic intervention studies.
- Translational validation in human cohorts is needed to assess clinical efficacy, pharmacokinetics, and long-term safety.
- In the present study, Oil Red O staining was primarily employed for morphological visualization of intracellular lipid accumulation rather than quantitative assessment. Absorbance-based quantification (OD510) was not performed in triplicate; therefore, the available data are insufficient for robust statistical analysis. This methodological limitation has been explicitly acknowledged in the revised Discussion section. Ongoing follow-up experiments include quantitative Oil Red O assays (≥3 biological replicates) to confirm the microscopic observations and strengthen evidence for the anti-steatotic effects of the BuOH fraction.

Nevertheless, cellular biochemical assays for TG and TC levels (Figures 8b,c) provide quantitative results consistent with the staining patterns, supporting the overall reliability of our findings.

Future research should therefore focus on dose–response and PK/PD characterization of SCH-derived lignans, functional validation of key molecular targets, and well-designed clinical trials to promote the translational application of Schisandra chinensis in NAFLD prevention and therapy.

## 5 Conclusion

In summary, our integrative study demonstrates that Schisandra chinensis exerts robust protective effects against NAFLD via multi-mechanistic actions. MR analysis establishes circulating TG as an independent causal risk factor for NAFLD and highlights lipid modulation as a key therapeutic axis. *In vivo*, SCH reduced weight gain, improved serum lipid profiles, attenuated hepatic steatosis, and restored liver function without affecting food intake. SCH restructured the gut microbiome—suppressing pro-inflammatory taxa and enriching beneficial microorganisms—and modulated bile acid and SCFA-related pathways, indicating gut–liver axis regulation. Hepatic lipidomics confirmed reprogramming of glycerophospholipid, glycerolipid and sphingolipid metabolism, while *in vitro* assays identified the BuOH fraction as the primary anti-steatotic fraction. Collectively, these findings support SCH as a multi-target candidate for NAFLD therapy; however, clinical validation is required.

## Data Availability

The original contributions presented in the study are publicly available. This data can be found here: NCBI SRA (BioProject: Accession PRJNA1380414; available at https://www.ncbi.nlm.nih.gov/sra/PRJNA1380414) and OMIX (ID OMIX013807; available at https://ngdc.cncb.ac.cn/omix/release/OMIX013807).
