# Supplementary material for: Schisandra chinensis alleviates Hypertriglyceridemia in nonalcoholic fatty liver disease by modulating the gut microbiota and hepatic lipid metabolism: identification of its active fractions
Source: Front Pharmacol. 2026 Jan 5;16:1715364. doi: 10.3389/fphar.2025.1715364 (PMC12813172; doi:10.3389/fphar.2025.1715364)
Supplement: Supplementary file 1 [file Table1.docx]

| Group | Treatment description | Diet type | Sex | Strain / species | Age (weeks) | Mean initial body weight (g, mean ± SD) | No. of animals (n) |
| --- | --- | --- | --- | --- | --- | --- | --- |
| NC | Normal control (saline) | Standard diet | Male | C57BL/6 mouse | 8 | 20.4 ± 0.8 | 8 |
| MC | Model control (saline, HFD-induced NAFLD) | High-fat diet | Male | C57BL/6 mouse | 8 | 20.3 ± 0.9 | 8 |
| DC | Schisandra chinensis (SCH) low-dose group (0.41 g/kg/d, preventive model) | Standard diet | Male | C57BL/6 mouse | 8 | 20.5 ± 0.7 | 8 |
| SCH | Schisandra chinensis high-dose group (1.23 g/kg/d, therapeutic model) | High-fat diet | Male | C57BL/6 mouse | 8 | 20.2 ± 0.8 | 8 |
| FBR | Positive control (fenofibrate, 61.5 mg/kg/d) | High-fat diet | Male | C57BL/6 mouse | 8 | 20.1 ± 0.9 | 8 |

Table S1. Characteristics of experimental animals used in this study (n = 8 per group)

Notes:

1.All mice were SPF-grade and obtained from an accredited animal facility.

2.Animals were housed under controlled environmental conditions (temperature 22–25 °C, humidity 50–60%, 12 h light/dark cycle).

3.Randomization and blinding were implemented during allocation and data analysis.

4.No mortality or unexpected behavioral changes occurred during the 12-week experiment.
